# Trends in cardiovascular disease incidence among 22 million people in the UK over 20 years: population based study

**DOI:** 10.1136/bmj-2023-078523

**Published:** 2024-06-26

**Authors:** Nathalie Conrad, Geert Molenberghs, Geert Verbeke, Francesco Zaccardi, Claire Lawson, Jocelyn M Friday, Huimin Su, Pardeep S Jhund, Naveed Sattar, Kazem Rahimi, John G Cleland, Kamlesh Khunti, Werner Budts, John J V McMurray

**Affiliations:** 1 School of Cardiovascular and Metabolic Health, British Heart Foundation Cardiovascular Research Centre, University of Glasgow, Glasgow, UK; 2 Department of Cardiovascular Sciences, KU Leuven, Leuven, Belgium; 3 Deep Medicine, Nuffield Department of Women’s and Reproductive Health, University of Oxford, Oxford, UK; 4 Interuniversity Institute for Biostatistics and statistical Bioinformatics (I-BioStat), Hasselt University and KU Leuven, Belgium; 5 Leicester Real World Evidence Unit, Diabetes Research Centre, University of Leicester, Leicester, UK; 6 College of Medical, Veterinary and Life Sciences, University of Glasgow, Glasgow, UK; 7 Congenital and Structural Cardiology, University Hospitals Leuven, Belgium

## Abstract

**Objective:**

To investigate the incidence of cardiovascular disease (CVD) overall and by age, sex, and socioeconomic status, and its variation over time, in the UK during 2000-19.

**Design:**

Population based study.

**Setting:**

UK.

**Participants:**

1 650 052 individuals registered with a general practice contributing to Clinical Practice Research Datalink and newly diagnosed with at least one CVD from 1 January 2000 to 30 June 2019.

**Main outcome measures:**

The primary outcome was incident diagnosis of CVD, comprising acute coronary syndrome, aortic aneurysm, aortic stenosis, atrial fibrillation or flutter, chronic ischaemic heart disease, heart failure, peripheral artery disease, second or third degree heart block, stroke (ischaemic, haemorrhagic, and unspecified), and venous thromboembolism (deep vein thrombosis or pulmonary embolism). Disease incidence rates were calculated individually and as a composite outcome of all 10 CVDs combined and were standardised for age and sex using the 2013 European standard population. Negative binomial regression models investigated temporal trends and variation by age, sex, and socioeconomic status.

**Results:**

The mean age of the population was 70.5 years and 47.6% (n=784 904) were women. The age and sex standardised incidence of all 10 prespecified CVDs declined by 19% during 2000-19 (incidence rate ratio 2017-19 *v* 2000-02: 0.80, 95% confidence interval 0.73 to 0.88). The incidence of coronary heart disease and stroke decreased by about 30% (incidence rate ratios for acute coronary syndrome, chronic ischaemic heart disease, and stroke were 0.70 (0.69 to 0.70), 0.67 (0.66 to 0.67), and 0.75 (0.67 to 0.83), respectively). In parallel, an increasing number of diagnoses of cardiac arrhythmias, valve disease, and thromboembolic diseases were observed. As a result, the overall incidence of CVDs across the 10 conditions remained relatively stable from the mid-2000s. Age stratified analyses further showed that the observed decline in coronary heart disease incidence was largely restricted to age groups older than 60 years, with little or no improvement in younger age groups. Trends were generally similar between men and women. A socioeconomic gradient was observed for almost every CVD investigated. The gradient did not decrease over time and was most noticeable for peripheral artery disease (incidence rate ratio most deprived *v* least deprived: 1.98 (1.87 to 2.09)), acute coronary syndrome (1.55 (1.54 to 1.57)), and heart failure (1.50 (1.41 to 1.59)).

**Conclusions:**

Despite substantial improvements in the prevention of atherosclerotic diseases in the UK, the overall burden of CVDs remained high during 2000-19. For CVDs to decrease further, future prevention strategies might need to consider a broader spectrum of conditions, including arrhythmias, valve diseases, and thromboembolism, and examine the specific needs of younger age groups and socioeconomically deprived populations.

## Introduction

Since the 1970s, the prevention of coronary disease, both primary and secondary, has improved considerably, largely attributable to public health efforts to control risk factors, such as antismoking legislation, and the widespread use of drugs such as statins.^1^ ^2^

Improvements in mortality due to heart disease have, however, stalled in several high income countries,^3^ and reports suggest that the incidence of heart disease might even be increasing among younger people.^4^ ^5^ ^6^ Conversely, along with coronary heart disease, other cardiovascular conditions are becoming relatively more prominent in older people, altering the profile of cardiovascular disease (CVD) in ageing societies. The importance of non-traditional risk factors for atherosclerotic diseases, such as socioeconomic deprivation, has also been increasingly recognised. Whether socioeconomic deprivation is as strongly associated with other CVDs as with atherosclerosis is uncertain, but it is important to understand as many countries have reported an increase in socioeconomic inequalities.^7^

Large scale epidemiological studies are therefore needed to investigate secular trends in CVDs to target future preventive efforts, highlight the focus for future clinical trials, and identify healthcare resources required to manage emerging problems. Existing comprehensive efforts, such as statistics on CVD from leading medical societies or the Global Burden of Diseases studies, have helped toward this goal, but reliable age standardised incidence rates for all CVDs, how these vary by population subgroups, and changes over time are currently not available.^8^ ^9^ ^10^

We used a large longitudinal database of linked primary care, secondary care, and death registry records from a representative sample of the UK population^11^ ^12^ to assess trends in the incidence of 10 of the most common CVDs in the UK during 2000-19, and how these differed by sex, age, socioeconomic status, and region.

## Methods

### Data source and study population

We used anonymised electronic health records from the GOLD and AURUM datasets of Clinical Practice Research Datalink (CPRD). CPRD contains information on about 20% of the UK population and is broadly representative of age, sex, ethnicity, geographical spread, and socioeconomic deprivation.^11^ ^12^ It is also one of the largest databases of longitudinal medical records from primary care in the world and has been validated for epidemiological research for a wide range of conditions.^11^ We used the subset of CPRD records that linked information from primary care, secondary care from Hospital Episodes Statistics (HES admitted patient care and HES outpatient) data, and death certificates from the Office for National Statistics (ONS). Linkage was possible for a subset of English practices, covering about 50% of the CPRD records. Data coverage dates were 1 January 1985 to 31 December 2019 for primary care data (including drug prescription data), 1 April 1997 to 30 June 2019 for secondary care data, and 2 January 1998 to 30 May 2019 for death certificates.

Included in the study were men and women registered with a general practice for at least one year during the study period (1 January 2000 to 30 June 2019) whose records were classified by CPRD as acceptable for use in research and approved for HES and ONS linkage.

### Study endpoints

The primary endpoint was the first presentation of CVD as recorded in primary or secondary care. We investigated 10 CVDs: acute coronary syndrome, aortic aneurysm, aortic stenosis, atrial fibrillation or flutter, chronic ischaemic heart disease, heart failure, peripheral artery disease, second or third degree heart block, stroke (ischaemic, haemorrhagic, or unspecified), and venous thromboembolism (deep vein thrombosis or pulmonary embolism). We defined incident diagnoses as the first record of that condition in primary care or secondary care regardless of its order in the patient’s record.

Diseases were considered individually and as a composite outcome of all 10 CVDs combined. For the combined analyses, we calculated the primary incidence (considering only the first recorded CVD in each patient, reflecting the number of patients affected by CVDs) and the total incidence (considering all incident CVD diagnoses in each patient, reflecting the cumulative number of CVD diagnoses). We performed sensitivity analyses including diagnoses recorded on death certificates.

To identify diagnoses, we compiled a list of diagnostic codes based on the coding schemes in use in each data source following previously established methods.^13^ ^14^ ^15^ We used ICD-10 (international classification of diseases, 10th revision) codes for diagnoses recorded in secondary care, ICD-9 (international classification of diseases, ninth revision) (in use until 31 December 2000) and ICD-10 codes for diagnoses recorded on death certificates (used in sensitivity analyses only), the UK Office of Population Censuses and Surveys classification (OPCS-4) for procedures performed in secondary care settings, and a combination of Read, SNOMED, and local EMIS codes for diagnoses recorded in primary care records (see supplementary table S1).^16^ Supplementary texts S1, S2, and S3 describe our approach to the generation of the diagnostic code list as well as considerations and sensitivity analyses into the validity of diagnoses recorded in UK electronic health records.

### Covariates

We selected covariates to represent a range of known cardiovascular risk factors. For clinical data, including systolic and diastolic blood pressure, smoking status, cholesterol (total:high density lipoprotein ratio), and body mass index (BMI), we abstracted data from primary care records as the most recent measurement within two years before the incident CVD diagnosis. BMI was categorised as underweight (<18.5), normal (18.5-24.9), overweight (25-29.9), and obesity (≥30). Information on the prevalence of chronic kidney disease, dyslipidaemia, hypertension, and type 2 diabetes was obtained as the percentage of patients with a diagnosis recorded in their primary care or secondary care record at any time up to and including the date of a first CVD diagnosis. Patients’ socioeconomic status was described using the index of multiple deprivation 2015,^17^ a composite measure of seven dimensions (income, employment, education, health, crime, housing, living environment) and provided by CPRD. Measures of deprivation are calculated at small area level, covering an average population of 1500 people, and are presented in fifths, with the first 20% and last 20% representing the least and most deprived areas, respectively. We extracted information on ethnicity from both primary and secondary care records, and we used secondary care data when records differed. Ethnicity was grouped into four categories: African/Caribbean, Asian, white, and mixed/other. Finally, we extracted information on cardiovascular treatments (ie, aspirin and other antiplatelets, alpha adrenoceptor antagonists, aldosterone antagonists/mineralocorticoid receptor antagonists, angiotensin converting enzyme inhibitors, angiotensin II receptor antagonists, beta blockers, calcium channel blockers, diuretics, nitrates, oral anticoagulants, and statins) as the number of patients with at least two prescriptions of each drug class within six months after incident CVD, among patients alive and registered with a general practitioner 30 days after the diagnosis. Supplementary table S2 provides a list of substances included in each drug class. Prescriptions were extracted from primary care records up to 31 December 2019.

### Statistical analyses

Categorical data for patient characteristics are presented as frequencies (percentages), and continuous data are presented as means and standard deviations (SDs) for symmetrically distributed data or medians and interquartile ranges (IQRs) for non-symmetrically distributed data, over the whole CVD cohort and stratified by age, sex, socioeconomic status, region, and calendar year of diagnosis. For variables with missing entries, we present numbers and percentages of records with missing data. For categorical variables, frequencies refer to complete cases.

Incidence rates of CVD were calculated by dividing the number of incident diagnoses by the number of patient years in the cohort. Category specific rates were computed separately for subgroups of age, sex, socioeconomic status, region, and calendar year of diagnosis. Age calculations were updated for each calendar year. To ensure calculations referred to incident diagnoses, we excluded individuals, from both the numerator and the denominator populations, with a disease of interest diagnosed before the study start date (1 January 2000), or within the first 12 months of registration with their general practice. Time at risk started at the latest of the patient’s registration date plus 12 months, 30 June of their birth year, or study start date; and stopped at the earliest of death, transfer out of practice, last collection date of the practice, incidence of the disease of interest, or linkage end date (30 June 2019). Disease incidence was standardised for age and sex^18^ using the 2013 European standard population^19^ in five year age bands up to age 90 years.

Negative binomial regression models were used to calculate overall and category specific incidence rate ratios and corresponding 95% confidence intervals (CIs).^20^ Models were adjusted for calendar year of diagnosis, age (categorised into five years age bands), sex, socioeconomic status, and region. We chose negative binomial models over Poisson models to account for potential overdispersion in the data. Sensitivity analyses comparing Poisson and negative binomial models showed similar results.

Study findings are reported according to the RECORD (reporting of studies conducted using observational routinely collected health data) recommendations.^21^ We performed statistical analyses in R, version 4.3.3 (R Foundation for Statistical Computing, Vienna, Austria).

### Patient and public involvement

No patients or members of the public were directly involved in this study owing to constraints on funding and time.

## Results

A total of 22 009 375 individuals contributed data between 1 January 2000 and 30 June 2019, with 146 929 629 patient years of follow-up. Among those we identified 2 906 770 new CVD diagnoses, affecting 1 650 052 patients. Mean age at first CVD diagnosis was 70.5 (SD 15.0) years, 47.6% (n=784 904) of patients were women, and 11.6% (n=191 421), 18.0% (n=296 554), 49.7% (n=820 892), and 14.2% (n=233 833) of patients had a history of chronic kidney disease, dyslipidaemia, hypertension, and type 2 diabetes, respectively, at the time of their first CVD diagnosis (table 1).

**Table 1:** Characteristics of patients with a first diagnosis of CVD, 2000-19. Values are number (percentage) unless stated otherwise

Characteristics	All patients (n=1 650 052)	Sex			Socioeconomic status*			Period	
		Women (n=784 904)	Men (n=865 148)		Least deprived (n=353 985)	Most deprived (n=299 230)		2000-02 (n=318 297)	2017-19 (n=194 908)
Age at first CVD diagnosis (years):									
Mean (SD)	70.5 (15.0)	73.2 (15.3)	68.2 (14.4)		71.8 (14.3)	67.9 (15.8)		71.5 (13.6)	69.5 (15.9)
Median (IQR)	73.0 (62.0-82.0)	76.0 (65.0-84.0)	70.0 (59.0-79.0)		74.0 (63.0-82.0)	70.0 (58.0-80.0)		74.0 (64.0-81.0)	71.0 (60.0-81.0)
Women	784 904 (47.6)	784 904 (100)	–		162 664 (46.0)	146 160 (48.8)		153 643 (48.3)	90 857 (46.6)
Ethnicity:									
African/Caribbean	25 518 (1.6)	12 595 (1.7)	12 923 (1.6)		1089 (0.3)	11572 (4.0)		2958 (1.0)	4600 (2.4)
Asian	41 704 (2.6)	17 418 (2.3)	24 286 (2.9)		5687 (1.7)	11278 (3.9)		4817 (1.7)	7527 (3.9)
Mixed/other	33 582 (2.1)	14 163 (1.9)	19 419 (2.3)		6960 (2.1)	6453 (2.2)		3823 (1.3)	7342 (3.8)
White	1 480 577 (93.6)	705 549 (94.1)	775 028 (93.2)		324 523 (95.9)	259 948 (89.9)		271 678 (95.9)	172 738 (89.9)
Missing	68 671 (4.2)	35 179 (4.5)	33 492 (3.9)		15 726 (4.4)	9979 (3.3)		35 021 (11.0)	2701 (1.4)
Socioeconomic level:									
1 (least deprived)	353 985 (21.5)	162 664 (20.7)	191 321 (22.1)		353 985 (100)	0 (0)		63 971 (20.1)	45 083 (23.1)
2	344 196 (20.9)	161 666 (20.6)	182 530 (21.1)		0 (0)	0 (0)		65 115 (20.5)	41 231 (21.2)
3	336 564 (20.4)	160 731 (20.5)	175 833 (20.3)		0 (0)	0 (0)		65 729 (20.7)	38 693 (19.9)
4	316 077 (19.2)	153 683 (19.6)	162 394 (18.8)		0 (0)	0 (0)		62 902 (19.8)	36 288 (18.6)
5 (most deprived)	299 230 (18.1)	146 160 (18.6)	153 070 (17.7)		0 (0)	299 230 (100)		60 580 (19.0)	33 613 (17.2)
Systolic blood pressure (mm Hg):									
Mean (SD)	138 (20.2)	139 (21.0)	138 (19.5)		138 (19.7)	137 (20.8)		146 (22.2)	134 (18.2)
Missing	256 402 (15.5)	108 110 (13.8)	148 292 (17.1)		51 473 (14.5)	51 046 (17.1)		104 686 (32.9)	19 751 (10.1)
Diastolic blood pressure (mm Hg):									
Mean (SD)	78.2 (11.5)	77.7 (11.4)	78.6 (11.6)		78.1 (11.3)	78.2 (11.8)		81.2 (11.4)	76.9 (11.4)
Missing	256 952 (15.6)	108 456 (13.8)	148 496 (17.2)		51 529 (14.6)	51 230 (17.1)		105 080 (33.0)	19 773 (10.1)
Body mass index category:									
Underweight	22 826 (2.9)	15 805 (4.2)	7021 (1.6)		4084 (2.5)	5354 (3.4)		2007 (2.4)	3229 (2.8)
Normal weight	228 699 (28.6)	115 615 (31.0)	113 084 (26.4)		50 028 (30.8)	42 313 (27.1)		25 326 (30.5)	29 581 (26.0)
Overweight	287 902 (36.0)	115 160 (30.9)	172 742 (40.4)		62 570 (38.5)	51 136 (32.7)		32 227 (38.7)	39 342 (34.6)
Obesity	261 058 (32.6)	126 134 (33.8)	134 924 (31.5)		45985 (28.3)	57 605 (36.8)		23 610 (28.4)	41 474 (36.5)
Missing	849 567 (51.5)	412 190 (52.5)	437 377 (50.6)		191 318 (54.0)	142 822 (47.7)		235 127 (73.9)	81 282 (41.7)
Smoking status:									
Current smoker	226 019 (21.3)	91 087 (18.5)	134 932 (23.7)		31 255 (14.2)	64 165 (31.7)		20 889 (23.9)	28 575 (20.6)
Former smoker	353 276 (33.3)	130 324 (26.5)	222 952 (39.1)		76109 (34.6)	60 989 (30.2)		24 326 (27.9)	48 189 (34.7)
Non-smoker	482 768 (45.5)	270 521 (55.0)	212 247 (37.2)		112431 (51.2)	77 078 (38.1)		42 102 (48.2)	62 184 (44.8)
Missing	587 989 (35.6)	292 972 (37.3)	295 017 (34.1)		134190 (37.9)	96 998 (32.4)		230 980 (72.6)	55 960 (28.7)
Cholesterol (total:HDL ratio):									
Mean (SD)	3.73 (1.26)	3.51 (1.16)	3.91 (1.30)		3.68 (1.22)	3.80 (1.31)		4.18 (1.57)	3.60 (1.21)
Missing	1 049256 (63.6)	513 320 (65.4)	535 936 (61.9)		220 408 (62.3)	196 779 (65.8)		302 541 (95.1)	82 721 (42.4)
History of comorbidities:									
Chronic kidney disease	191 421 (11.6)	107 974 (13.8)	83 447 (9.6)		40 967 (11.6)	34 441 (11.5)		5296 (1.7)	36 366 (18.7)
Dyslipidaemia	296 554 (18.0)	136 879 (17.4)	159 675 (18.5)		62 020 (17.5)	58 069 (19.4)		26 942 (8.5)	54 488 (28.0)
Hypertension	820 892 (49.7)	418 826 (53.4)	402 066 (46.5)		176 523 (49.9)	149 492 (50.0)		122 452 (38.5)	113 822 (58.4)
Type 2 diabetes	233 833 (14.2)	103 260 (13.2)	130 573 (15.1)		42 315 (12.0)	51 107 (17.1)		30 049 (9.4)	37 416 (19.2)

Blood pressure, body mass index, smoking status, and cholesterol level represent the latest measurement within two years before a first CVD diagnosis. Category percentages refer to complete cases. Supplementary table S3 presents patient characteristics stratified by age at first CVD diagnosis.

CVD=cardiovascular disease; HDL=high density lipoprotein; IQR=interquartile range; SD=standard deviation.

* Defined using index of multiple deprivation 2015.

**Fig 1:**
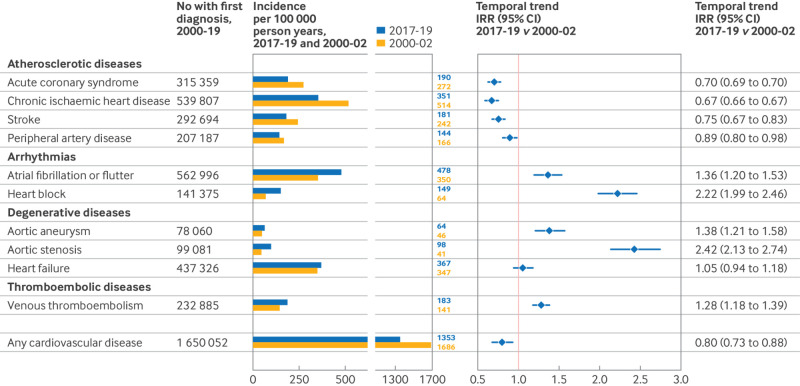
Incidence of a first diagnosis of cardiovascular disease per 100 000 person years, 2000-19. Incidence rates are age-sex standardised to the 2013 European standard population. Any cardiovascular disease refers to the primary incidence of cardiovascular disease across the10 conditions investigated (ie, number of patients with a first diagnosis of cardiovascular disease). See supplementary table S4 for crude incidence rates by age and sex groups. IRR=incidence rate ratio

During 2017-19, the most common CVDs were atrial fibrillation or flutter (age-sex standardised incidence 478 per 100 000 person years), heart failure (367 per 100 000 person years), and chronic ischaemic heart disease (351 per 100 000 person years), followed by acute coronary syndrome (190 per 100 000 person years), venous thromboembolism (183 per 100 000 person years), and stroke (181 per 100 000 patient years) (fig 1).

### Temporal trends

The primary incidence of CVDs (ie, the number of patients with CVD) decreased by 20% during 2000-19 (age-sex standardised incidence rate ratio 2017-19 *v* 2000-02: 0.80 (95% CI 0.73 to 0.88)). However, the total incidence of CVD (ie, the total number of new CVD diagnoses) remained relatively stable owing to an increasing number of subsequent diagnoses among patients already affected by a first CVD (incidence rate ratio 2017-19 *v* 2000-02: 1.00 (0.91 to 1.10)).

The observed decline in CVD incidence was largely due to declining rates of atherosclerotic diseases, in particular acute coronary syndrome, chronic ischaemic heart disease, and stroke, which decreased by about 30% during 2000-19. The incidence of peripheral artery disease also declined, although more modestly (incidence rate ratio 2017-19 *v* 2000-02: 0.89 (0.80 to 0.98)) (fig 1).

**Fig 2:**
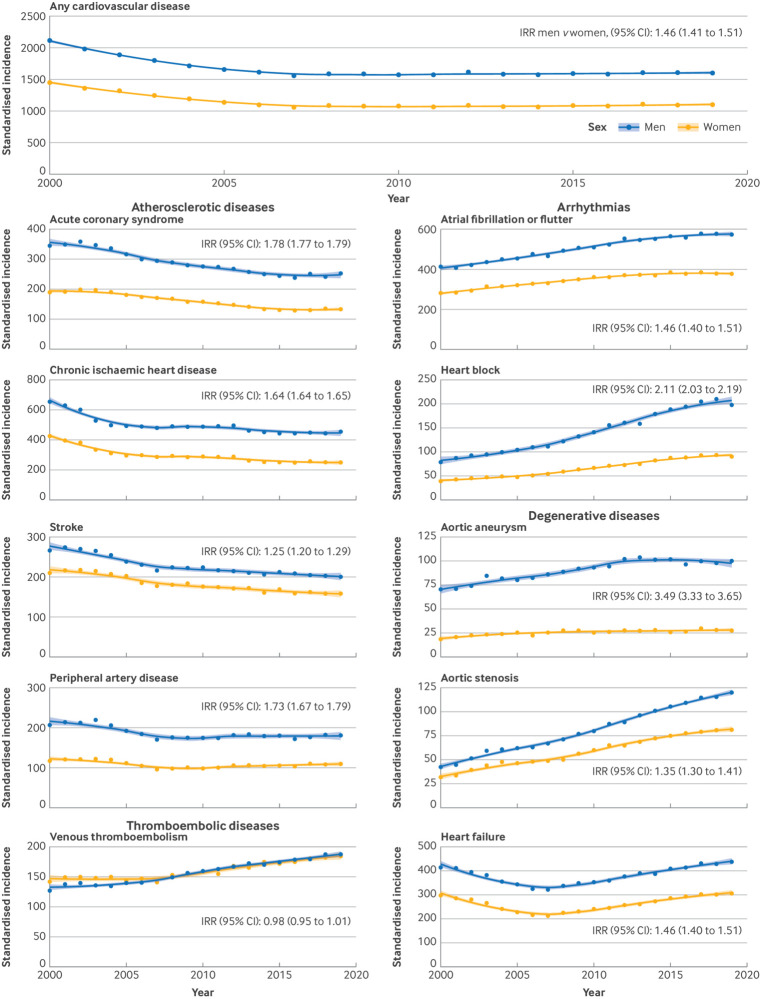
Age standardised incidence of cardiovascular disease by sex, 2000-19. Any cardiovascular disease refers to the primary incidence of cardiovascular disease across the 10 conditions investigated (ie, number of patients with a first diagnosis of cardiovascular disease). IRR=incidence rate ratio

The incidence of non-atherosclerotic heart diseases increased at varying rates, with incidence of aortic stenosis and heart block more than doubling over the study period (2017-19 *v* 2000-02: 2.42 (2.13 to 2.74) and 2.22 (1.99 to 2.46), respectively) (fig 1). These increasing rates of non-atherosclerotic heart diseases balanced the reductions in ischaemic diseases so that the overall incidence of CVD across the 10 conditions appeared to reach a plateau and to remain relatively stable from 2007-08 (incidence rate ratio 2017-19 *v* 2005-07: 1.00 (0.91 to 1.10)) (fig 2).

**Fig 3:**
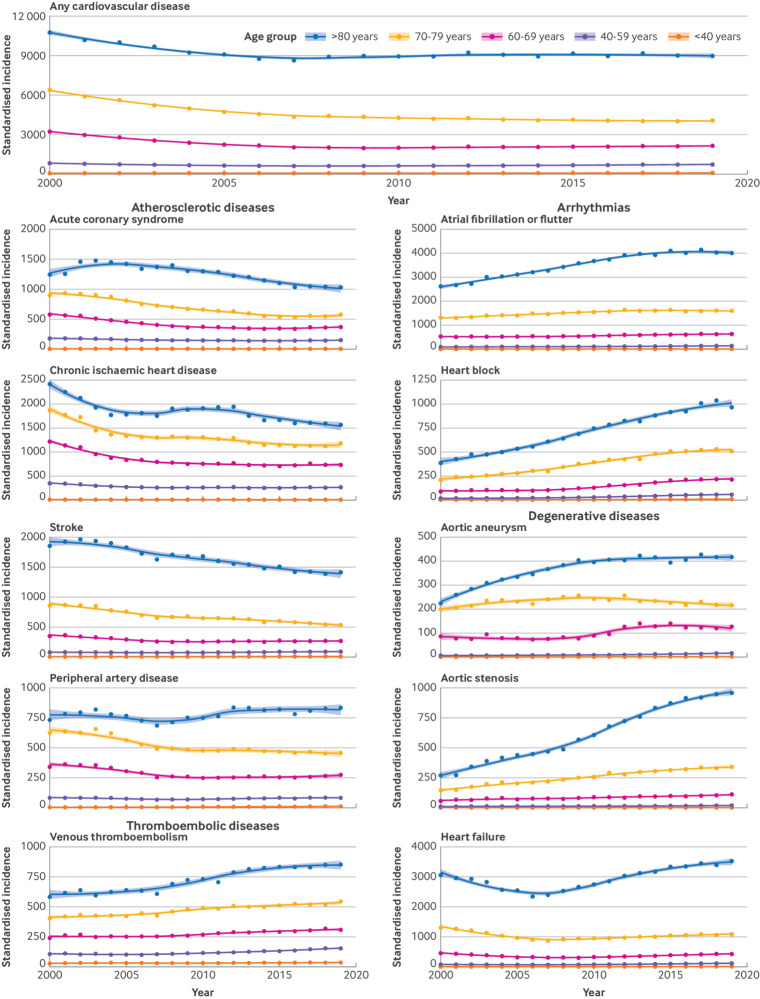
Sex standardised incidence of cardiovascular disease in all age groups. Any cardiovascular disease refers to the primary incidence of cardiovascular disease across the 10 conditions investigated (ie, number of patients with a first diagnosis of cardiovascular disease)

**Fig 4:**
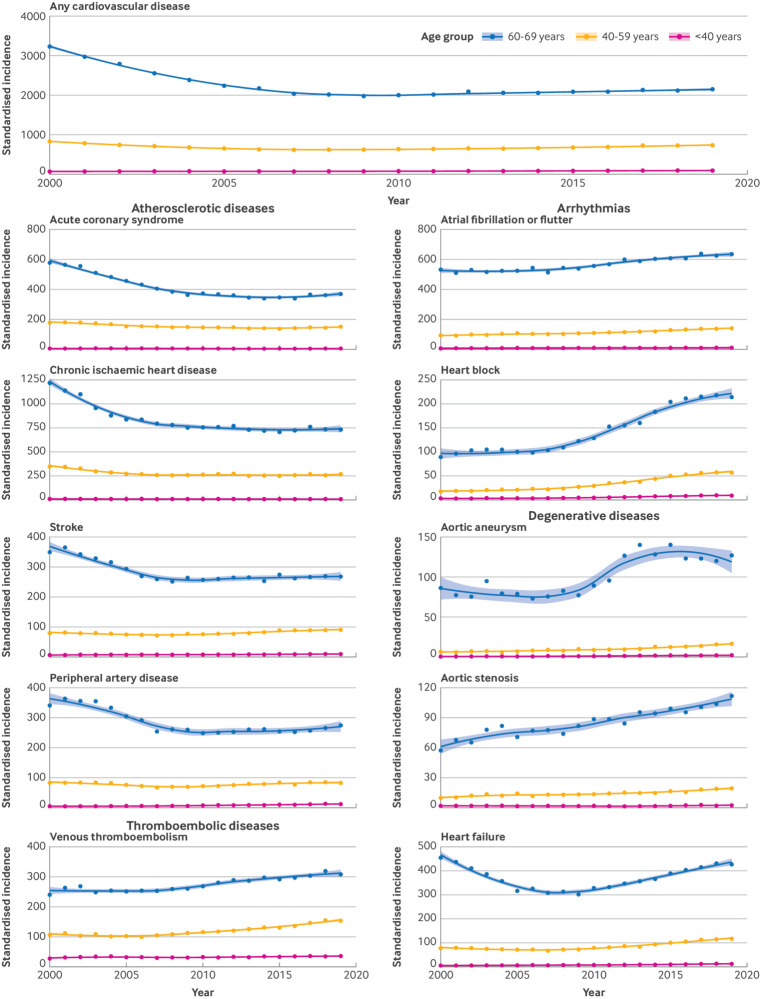
Sex standardised incidence of cardiovascular diseases by age subgroups <69 years. Any cardiovascular disease refers to the primary incidence of cardiovascular disease across the 10 conditions investigated (ie, number of patients with a first diagnosis of cardiovascular disease)

Age stratified analyses further showed that the observed decrease in incidence of chronic ischaemic heart disease, acute coronary syndrome, and stroke was largely due to a reduced incidence in those aged >60 years, whereas incidence rates in those aged <60 years remained relatively stable (fig 3 and fig 4).

### Age at diagnosis

**Fig 5:**
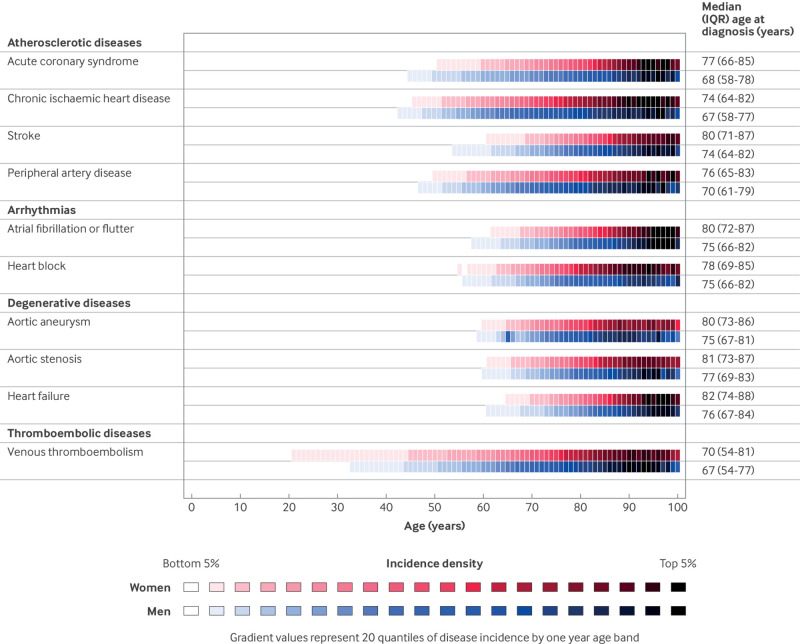
Incidence rates of cardiovascular diseases calculated by one year age bands and divided into a colour gradient of 20 quantiles to reflect incidence density by age. IQR=interquartile range

CVD incidence was largely concentrated towards the end of the life span, with a median age at diagnosis generally between 65 and 80 years. Only venous thromboembolism was commonly diagnosed before age 45 years (fig 5). Over the study period, age at first CVD diagnosis declined for several conditions, including stroke (on average diagnosed 1.9 years earlier in 2019 than in 2000), heart block (1.3 years earlier in 2019 than in 2000), and peripheral artery disease (1 year earlier in 2019 than in 2000) (see supplementary figure S1). Adults with a diagnosis before age 60 years were more likely to be from lower socioeconomic groups and to have a higher prevalence of several risk factors, including obesity, smoking, and high cholesterol levels (see supplementary table S3).

### Incidence by sex

Age adjusted incidence of all CVDs combined was higher in men (incidence rate ratio for women *v* men: 1.46 (1.41 to 1.51)), with the notable exception of venous thromboembolism, which was similar between men and women. The incidence of aortic aneurysms was higher in men (3.49 (3.33 to 3.65)) (fig 2). The crude incidence of CVD, however, was similar between men and women (1069 per 100 000 patient years and 1176 per 100 000 patient years, respectively), owing to the higher number of women in older age groups. Temporal trends in disease incidence were generally similar between men and women (fig 2).

### Incidence by socioeconomic status

**Fig 6:**
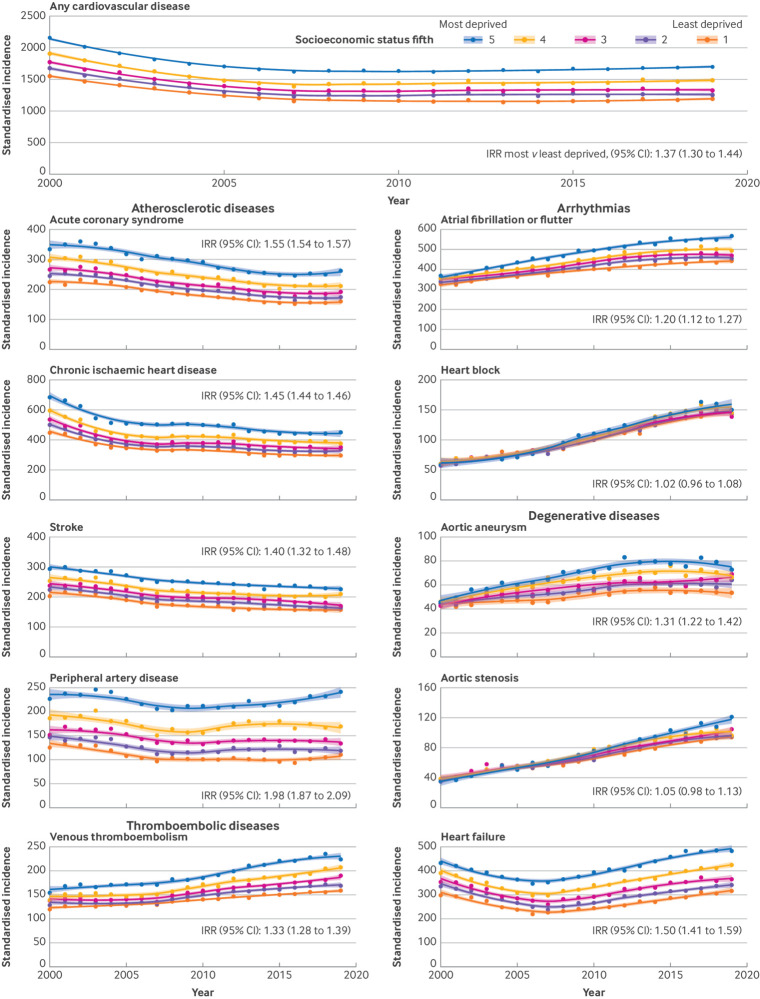
Age-sex standardised incidence rates of cardiovascular diseases by socioeconomic status (index of multiple deprivation 2015). Any cardiovascular disease refers to the primary incidence of cardiovascular disease across the 10 conditions investigated (ie, number of patients with a first diagnosis of cardiovascular disease). Yearly incidence estimates were smoothed using loess (locally estimated scatterplot smoothing) regression lines

The most deprived socioeconomic groups had a higher incidence of any CVDs (incidence rate ratio most deprived *v* least deprived: 1.37 (1.30 to 1.44)) (fig 6). A socioeconomic gradient was observed across almost every condition investigated. That gradient did not decrease over time, and it was most noticeable for peripheral artery disease (incidence rate ratio most deprived *v* least deprived: 1.98 (1.87 to 2.09)), acute coronary syndrome (1.55 (1.54 to 1.57)), and heart failure (1.50 (1.41 to 1.59)). For aortic aneurysms, atrial fibrillation, heart failure, and aortic stenosis, socioeconomic inequalities in disease incidence appeared to increase over time.

### Regional differences

Higher incidence rates were seen in northern regions (north west, north east, Yorkshire and the Humber) of England for all 10 conditions investigated, even after adjusting for socioeconomic status. Aortic aneurysms and aortic stenosis had the strongest regional gradients, with incidence rates about 30% higher in northern regions compared with London. Geographical variations remained modest, however, and did not appear to change considerably over time (see supplementary figure S2).

### Sensitivity analyses

In sensitivity analyses that used broader disease definitions, that included diagnoses recorded on death certificates, that relied on longer lookback periods for exclusion of potentially prevalent diagnoses, or that were restricted to diagnoses recorded during hospital admissions, temporal trends in disease incidence appeared similar (see supplementary figures S3-S6).

### Secondary prevention treatments

The proportion of patients using statins and antihypertensive drugs after a first CVD diagnosis increased over time, whereas the use of non-dihydropyridines calcium channel blockers, nitrates, and diuretics decreased over time. Non-vitamin K antagonist oral anticoagulants increasingly replaced vitamin K anticoagulants (see supplementary figure S7).

## Discussion

The findings of this study suggest that important changes occurred in the distribution of CVDs during 2000-19 and that several areas are of concern. The incidence of non-atherosclerotic heart diseases was shown to increase, the decline in atherosclerotic disease in younger people was stalling, and socioeconomic inequalities had a substantial association across almost every CVD investigated.

### Implications for clinical practice and policy

Although no causal inference can be made from our data, the decline in rates of ischaemic diseases coincided with reductions in the prevalence of risk factors such as smoking, hypertension, and raised cholesterol levels in the general population over the same period,^22^ and this finding suggests that efforts in the primary and secondary prevention of atherosclerotic diseases have been successful. The decline in stroke was not as noticeable as that for coronary heart disease, which may reflect the rising incidence of atrial fibrillation. The variation in trends for peripheral artery disease could be due to differences in risk factors (eg, a stronger association with diabetes), the multifaceted presentations and causes, and the introduction of systematic leg examinations for people with diabetes.^23^ ^24^

All the non-atherosclerotic diseases, however, appeared to increase during 2000-19. For some conditions, such as heart failure, the observed increase remained modest, whereas for others, such as aortic stenosis and heart block, incidence rates doubled. All analyses in this study were standardised for age and sex, to illustrate changes in disease incidence independently of changes in population demographics. Whether these trends solely reflect increased awareness, access to diagnostic tests, or even screening (eg, for abdominal aortic aneurysm^25^) and coding practices, is uncertain. Reductions in premature death from coronary heart disease may have contributed to the emergence of these other non-atherosclerotic CVDs. Regardless, the identification of increasing numbers of people with these problems has important implications for health services, especially the provision of more surgical and transcatheter valve replacement, pacemaker implantation, and catheter ablation for atrial fibrillation. Importantly, these findings highlight the fact that for many cardiovascular conditions such as heart block, aortic aneurysms, and non-rheumatic valvular diseases, current medical practice remains essentially focused on the management of symptoms and secondary prevention and that more research into underlying causes and possible primary prevention strategies is needed.^26^ ^27^

These varying trends also mean that the contribution of individual CVDs towards the overall burden has changed. For example, atrial fibrillation or flutter are now the most common CVDs in the UK. Atrial fibrillation is also a cause (and consequence) of heart failure, and these two increasingly common problems may amplify the incidence of each other. Venous thromboembolism and heart block also appeared as important contributors to overall CVD burden, with incidence rates similar to those of stroke and acute coronary syndrome, yet both receive less attention in terms of prevention efforts.

The stalling decline in the rate of coronary heart disease in younger age groups is of concern, has also been observed in several other high income countries, and may reflect rising rates of physical inactivity, obesity, and type 2 diabetes in young adults.^4^ ^6^ ^28^ The stalled decline suggests prevention approaches may need to be expanded beyond antismoking legislation, blood pressure control, and lipid lowering interventions to include the promotion of physical activity, weight control, and use of new treatments shown to reduce cardiovascular risk in people with type 2 diabetes.^29^ Although CVD incidence is generally low in people aged <60 years, identifying those at high risk of developing CVD at a young age and intervening before problems occur could reduce premature morbidity and mortality and have important economic implications.

Our study further found that socioeconomic inequalities may contribute to CVD burden, and that this association is not restricted to selected conditions but is visible across most CVDs. The reasons behind the observed increase in risk in relation to socioeconomic inequalities are likely to be multifactorial and to include environmental, occupational, psychosocial, and behavioural risk factors, including established cardiovascular risk factors such as smoking, obesity, nutrition, air pollution, substance misuse, and access to care.^30^ How these findings apply to different countries is likely to be influenced by socioeconomic structures and healthcare systems, although health inequalities have been reported in numerous countries.^30^ One important factor in the present study is that access to care is free at the point of care in the UK,^31^ and yet socioeconomic inequalities persist despite universal health coverage and they did not appear to improve over time. Independently of the specificities of individual countries, our findings highlight the importance of measuring and considering health inequalities and suggest that dealing with the social determinants of health—the conditions under which people are born, live, work, and age—could potentially bring substantial health improvements across a broad range of chronic conditions.

Finally, our results reflect disease incidence based on diagnostic criteria, screening practices, availability, and accuracy of diagnostic tests in place at a particular time and therefore must be interpreted within this context.^32^ Several of the health conditions investigated are likely to being sought and detected with increased intensity over the study period. For example, during the study period the definition of myocardial infarction was revised several times,^33^ ^34^ ^35^ and high sensitivity troponins were progressively introduced in the UK from 2010. These more sensitive markers of cardiac injury are thought to have increased the detection rates for less severe disease.^36^ ^37^ Similarly, increased availability of computed tomography may have increased detection rates for stroke.^38^ These changes could have masked an even greater decline in these conditions than observed in the present study. Conversely, increased use of other biochemical tests (such as natriuretic peptides) and more sensitive imaging techniques might have increased the detection of other conditions.^39^ ^40^ ^41^ The implementation of a screening programme for aortic aneurysm and incentive programmes aimed at improving coding practices, including the documentation of CVD, associated risk factors and comorbidities, and treatment of these, are also likely to have contributed to the observed trends.^25^ ^42^ ^43^ As a result, the difference in incidence estimates and prevalence of comorbidities over time may not reflect solely changes in the true incidence but also differences in ascertainment of people with CVD.^44^ Nonetheless, long term trends in large and unconstrained populations offer valuable insights for healthcare resource planning and for the design of more targeted prevention strategies that could otherwise not be answered by using smaller cohorts, cross sectional surveys, or clinical trials; and precisely because they are based on routinely reported diagnoses they are more likely to capture the burden of disease as experienced by doctors and health services.

### Strengths and limitations of this study

A key strength of this study is its statistical power, with >140 million person years of data. The large size of the cohort allowed us to perform incidence calculations for a broad spectrum of conditions, and to examine the influence of age, sex, and socioeconomic status as well as trends over 20 years. One important limitation of our study was the modest ethnic diversity in our cohort and the lack of information on ethnicity for the denominator population, which precluded us from stratifying incidence estimates by ethnic group. Our analyses were also limited by the unavailability or considerable missingness of additional variables potentially relevant to the development of CVD, such as smoking, body mass index, imaging data, women specific cardiovascular risk factors (eg, pregnancy associated hypertension and gestational diabetes), and blood biomarkers. Further research may also need to consider an even wider spectrum of CVDs, including individual types of valve disease, pregnancy related conditions, and infection related heart diseases. Research using databases with electronic health records is also reliant on the accuracy of clinical coding input by doctors in primary care as part of a consultation, or in secondary care as part of a hospital admission. We therefore assessed the validity of diagnoses in UK electronic health records data and considered it to be appropriate in accordance with the >200 independent validation studies reporting an average positive predictive value of about 90% for recorded diagnoses.^45^ Observed age distributions were also consistent with previous studies and added to the validity of our approach. Nevertheless, our results must be interpreted within the context and limitations of routinely collected data from health records, diagnostic criteria, screening practices, the availability and accuracy of diagnostic tests in place at that time, and the possibility that some level of miscoding is present or that some bias could have been introduced by restricting the cohort to those patients with at least 12 months of continuous data.

### Conclusions

Efforts to challenge the notion of the inevitability of vascular events with ageing, and evidence based recommendations for coronary heart disease prevention, have been successful and can serve as a model for other non-communicable diseases. Our findings show that it is time to expand efforts to improve the prevention of CVDs. Broadening research and implementation efforts in both primary and secondary prevention to non-atherosclerotic diseases, tackling socioeconomic inequalities, and introducing better risk prediction and management among younger people appear to be important opportunities to tackle CVDs.

**What is already known on this topic**

Recent data show that despite decades of declining rates of cardiovascular mortality, the burden from cardiovascular disease (CVD) appears to have stalled in several high income countries

**What this study adds**

This observational study of a representative sample of 22 million people from the UK during 2000-19 found reductions in CVD incidence to have been largely restricted to ischaemic heart disease and stroke, and were paralleled by a rising number of diagnoses of cardiac arrhythmias, valve disease, and thromboembolic events
Venous thromboembolism and heart block were important contributors to the overall burden of CVDs, with incidence rates similar to stroke and acute coronary syndromes
Improvements in rates of coronary heart disease almost exclusively appeared to benefit those aged >60 years, and the CVD burden in younger age groups appeared not to improve

## Data Availability

Access to Clinical Practice Research Datalink (CPRD) data is subject to a license agreement and protocol approval process that is overseen by CPRD’s research data governance process. A guide to access is provided on the CPRD website (https://www.cprd.com/data-access) To facilitate the subsequent use and replication of the findings from this study, aggregated data tables are provided with number of events and person years at risk by individual condition and by calendar year, age (by five year age band), sex, socioeconomic status, and region (masking field with fewer than five events, as per CPRD data security and privacy regulations) on our GitHub repository (https://github.com/nathalieconrad/CVD_incidence).

## Web extra

Extra material supplied by authors

## Notes

Contributors: NC and JJVM conceived and designed the study. NC, JJVM, GM, and GV designed the statistical analysis plan and NC performed the statistical analysis. All authors contributed to interpreting the results, drafting the manuscript, and the revisions. NC, GM, and GV had permission to access the raw data and NC and GM verified the raw data. All authors gave final approval of the version to be published and accept responsibility to submit the manuscript for publication. NC and JJVM accept full responsibility for the conduct of the study, had access to aggregated data, and controlled the decision to publish. They are the guarantors. The corresponding author attests that all listed authors meet authorship criteria and that no others meeting the criteria have been omitted.

Transparency: The lead author (NC) affirms that the manuscript is an honest, accurate, and transparent account of the study being reported; that no important aspects of the study have been omitted; and that any discrepancies from the study as planned (and, if relevant, registered) have been explained.

Dissemination to participants and related patient and public communities: Results from this study will be shared with patient associations and foundations dedicated to preventing cardiovascular diseases, such as the European Heart Network and the American Heart Association. To reach the public, findings will also be press released alongside publication of this manuscript. Social media (eg, X) will be used to draw attention to the work and stimulate debate about its findings. Finally, the underlying developed algorithms will be freely available for academic use at https://github.com/nathalieconrad/CVD_incidence.

Provenance and peer review: Not commissioned; externally peer reviewed.
