# Supplementary material for: Trends in cardiovascular disease incidence among 22 million people in the UK over 20 years: population based study
Source: BMJ. 2024 Jun 26;385:e078523. doi: 10.1136/bmj-2023-078523 (PMC11203392; doi:10.1136/bmj-2023-078523)
Supplement: Supplementary file 1 — Supplementary information: Supplementary text S1-S3, figures S1-S7, and tables S1-S4 [file conn078523.ww.pdf]

Temporal trends and patterns in cardiovascular disease incidence. A population-based study in 22 million individuals.

Supplementary Material

Supplementary text S1: Validity of diagnoses recorded in UK electronic health records 2

Supplementary text S2: Approach to diagnostic code list generation 3

Supplementary text S3: Examples of disease-specific sensitivity analyses investigating the validity of disease definitions 5

Figure S1: Temporal trends in age at first diagnosis of cardiovascular diseases 6

Figure S2: Incidence of cardiovascular diseases by region 7

Figure S3: Incidence of cardiovascular diseases over time from 2000-2019. Sensitivity analyses based on broader disease definitions. 8

Figure S4: Incidence of cardiovascular diseases over time from 2000-2019. Sensitivity analyses including diagnoses recorded on death certificates. 9

Figure S5: Incidence of cardiovascular diseases over time from 2000-2019. Sensitivity analyses with longer lookback period 10

Figure S6: Incidence of cardiovascular diseases over time from 2000-2019. Sensitivity analyses restricted to diagnoses recorded during hospital admissions. 11

Figure S7: Initiation of cardiovascular prevention therapy within 6 months of diagnosis, among patients diagnosed with cardiovascular disease in the periods 2000-2002 and 2017-2019. 12

Table S1: Clinical codes used to define cardiovascular diseases 13

Table S2: Drug class definitions 14

Table S3: Characteristics of patients with incident cardiovascular disease between 2000 and 2019, stratified by age at diagnosis 15

Table S4: Crude incidence rates of individual cardiovascular diseases, stratified by age and sex, for the period 2017-2019 16

Supplementary text S1: Validity of diagnoses recorded in UK electronic health records

Research using electronic health records databases is reliant on the accuracy of clinical coding input by physicians in primary care, as part of a consultation, or secondary care, as part of a hospital admission. The validity of diagnoses underlying our study has therefore been carefully assessed and was considered appropriate and supported by a solid evidence base.

To date, over 200 independent validation studies have been performed in the dataset underlying our study (CPRD) and these report an average positive predictive value of about 90% for a broad range of conditions. For cardiovascular outcomes specifically, over 50 validation studies exist,[1] including for the conditions investigated in our study, such as aortic stenosis, atrial fibrillation[2,3], heart failure[4], myocardial infarction[5,6], stroke[7], or venous thromboembolism[8], and many of these validation studies were directly performed in the dataset underlying our study (CPRD). As a result of this intensive research on data completeness and accuracy, research using CPRD data has informed drug safety guidance and clinical practice, and with over 3,000 peer-reviewed publications, it is generally agreed to be one of the most robust and best-studied routinely collected medical data sources in the world.[9,10]

In addition to the evidence from independent validation studies, we have compared disease incidence rates and trends over time with the literature and national healthcare audits, and performed careful validation and calibration of diagnostic code lists (details outlined in **Supplementary text S2**). We further performed sensitivity analyses using broader disease definitions (**Figure S3**), including diagnoses recorded on death certificates (**Figure S4**), using longer lookback periods (**Figure S5**), or restricting diagnoses recorded by specialists during hospital admission (**Figure S6**), and performed a series of disease-specific investigations into the validity of recorded diagnoses (**Supplementary text S3**). We found all of these to support the robustness and validity of the present data.

References:

1 Herrett E, Thomas SL, Schoonen WM, Smeeth L, Hall AJ. Validation and validity of diagnoses in the General Practice Research Database: a systematic review. British journal of clinical pharmacology 2010; 69: 4–14.

2 de Burgos-Lunar C, del Cura-González I, Cárdenas-Valladolid J, et al. Real-world data in primary care: validation of diagnosis of atrial fibrillation in primary care electronic medical records and estimated prevalence among consulting patients'. BMC Prim Care 2023; 24: 4.

3 Ruigómez A, Johansson S, Wallander M-A, Rodríguez LAG. Incidence of chronic atrial fibrillation in general practice and its treatment pattern. Journal of Clinical Epidemiology 2002; 55: 358–63.

4 Schaufelberger M, Ekestubbe S, Hultgren S, et al. Validity of heart failure diagnoses made in 2000–2012 in western Sweden. ESC Heart Fail 2019; 7: 36–45.

5 Herrett E, Shah AD, Boggon R, et al. Completeness and diagnostic validity of recording acute myocardial infarction events in primary care, hospital care, disease registry, and national mortality records: cohort study. BMJ (Clinical research ed) 2013; 346: f2350.

6 Hammad TA, McAdams MA, Feight A, Iyasu S, Dal Pan GJ. Determining the predictive value of Read/OXMIS codes to identify incident acute myocardial infarction in the General Practice Research Database. Pharmacoepidemiology and Drug Safety 2008; 17: 1197–201.

7 Woodfield R, Grant I, Group UBSO, Group UBF-U and OW, Sudlow CLM. Accuracy of Electronic Health Record Data for Identifying Stroke Cases in Large-Scale Epidemiological Studies: A Systematic Review from the UK Biobank Stroke Outcomes Group. PLOS ONE 2015; 10: e0140533.

8 Lawrenson R, Todd J-C, Leydon GM, Williams TJ, Farmer RDT. Validation of the diagnosis of venous thromboembolism in general practice database studies. British Journal of Clinical Pharmacology 2000; 49: 591–6.

9 Bibliography | CPRD. 2023; published online Jan 11. https://www.cprd.com/bibliography (accessed Feb 2, 2023).

10 Oyinlola JO, Campbell J, Kousoulis AA. Is real world evidence influencing practice? A systematic review of CPRD research in NICE guidances. BMC Health Services Research 2016; 16: 299.

Supplementary text S2: Approach to diagnostic code list generation

To establish the diagnostic code lists used in the present study, we have established and followed the approach detailed below. First, we filtered diagnostic and procedure code dictionaries for a broad set of keywords and synonyms that reflect the condition of interest. Second, two independent clinicians selected relevant codes, under consideration of the study's research question and special requirements for specificity and sensitivity. A third independent expert was consulted to resolve disagreements.

Following that, we performed careful validation and calibration of diagnostic code lists through the following steps:

- We compared and complemented diagnostic codes lists based on previous literature[1-12], online clinical code repositories (eg. Opensafely[13], BHF Data Science Centre[14], or HDR UK phenotype libraries[15]), and codes used in national healthcare audits[16-18], to ensure completeness.
- For each code, we extracted the number and frequency of occurrences within the population of interest and examined trends over time and differences between individual data sources (primary care, secondary care, death certificates and/or others).
- For every condition, we compared calculated disease incidence rates and trends over time with the literature[1-4,6-12, 16-29], and investigated methodological differences and clinical implications.
- Finally, we performed a range of sensitivity analyses to assess the robustness of incidence calculations to changes in disease definitions, eg. by focusing on specific disease subtypes (eg. ischaemic stroke), broadening disease definitions (eg. cerebrovascular diseases), or restricting analyses to a more specific set of diagnostic codes.

For the present study, the disease definition panel included cardiologists, primary care physicians, epidemiologists, and health services researchers – all with extensive expertise in UK healthcare systems and electronic health record studies. Specifically, the disease definition panel consisted of Prof. John McMurray, Prof. John Cleland, Prof. Naveed Sattar, Prof. Kazem Rahimi, Prof. Kamlesh Khunti and Dr. Nathalie Conrad. The clinical specialists on the panel are directly involved in treating patients with these conditions in the UK, both in primary care and secondary care settings; many have done so for over 30 years now and have personally witnessed changes in coding practices over the study period. By consensus, when designing this study, we chose disease definitions designed to optimise sensitivity and specificity i.e., unlikely to miss a substantial number of cases, but sufficiently restrictive to give valid estimates of incidence rates.

References:

1 Hippisley-Cox J, Coupland C, Brindle P. Development and validation of QRISK3 risk prediction algorithms to estimate future risk of cardiovascular disease: prospective cohort study. BMJ (Clinical research ed) 2017; 357: j2099.

2 Shah AD, Langenberg C, Rapsomaniki E, et al. Type 2 diabetes and incidence of cardiovascular diseases: a cohort study in 1·9 million people. The lancet Diabetes & endocrinology 2014; 8587: 1–9.

3 Tran J, Norton R, Conrad N, et al. Patterns and temporal trends of comorbidity among adult patients with incident cardiovascular disease in the UK between 2000 and 2014: A population-based cohort study. PLOS Medicine 2018; 15: e1002513.

4 Conrad N, Verbeke G, Molenberghs G, et al. Autoimmune diseases and cardiovascular risk: a population-based study on 19 autoimmune diseases and 12 cardiovascular diseases in 22 million individuals in the UK. Lancet (London, England) 2022; 0. DOI:10.1016/S0140-6736(22)01349-6.

5 UK Biobank Data Analyst and Scientific teams. UK Biobank, Algorithmically- defined outcomes (ADOs). Version 2.0 January 2022. https://biobank.ndph.ox.ac.uk/showcase/showcase/docs/alg_outcome_main.pdf

6 Martinsson A, Li X, Andersson C, Nilsson J, Smith JG, Sundquist K. Temporal Trends in the Incidence and Prognosis of Aortic Stenosis. Circulation 2015; 131: 988–94.

7 Andell P, Li X, Martinsson A, et al. Epidemiology of valvular heart disease in a Swedish nationwide hospital-based register study. Heart (British Cardiac Society) 2017; 103: 1696–703.

8 Wu J, Nadarajah R, Nakao YM, et al. Temporal trends and patterns in atrial fibrillation incidence: A population-based study of 3·4 million individuals. The Lancet Regional Health - Europe 2022; 17: 100386.

9 Christensen DM, Strange JE, Phelps M, et al. Age- and sex-specific trends in the incidence of myocardial infarction in Denmark, 2005 to 2021. Atherosclerosis 2022; 346: 63–7.

10 Chi GC, Kanter MH, Li BH, et al. Trends in Acute Myocardial Infarction by Race and Ethnicity. Journal of the American Heart Association 2020; 9: e013542.

11 Conrad N, Judge A, Tran J, et al. Temporal trends and patterns in heart failure incidence: a population-based study of 4 million individuals. The Lancet 2017; 391: 572–80.

12 Cea-Soriano L, Fowkes FGR, Johansson S, Allum AM, Rodriguez LAG. Time trends in peripheral artery disease incidence, prevalence and secondary preventive therapy: a cohort study in The Health Improvement Network in the UK. BMJ Open 2018; 8: e018184.

13 OpenSAFELY consortium. OpenSAFELY documentation and codelists. https://docs.opensafely.org/codelist-intro

14 BHF Data Science Centre. BHF Data Science Centre, diagnostic code list repository. GitHub. https://github.com/BHFDSC

15 National Institute for Health Data Science. HDR UK Phenotype Library. http://phenotypes.healthdatagateway.org

16 NHS Digital. Quality and Outcomes Framework (QOF) 2018-19. Data tables and indicator definitions. https://digital.nhs.uk/data-and-information/publications/statistical/quality-and-outcomes-framework-achievement-prevalence-and-exceptions-data/2018-19-pas

17 NHS Digital. Quality and Outcomes Framework (QOF) 2006-07. Data tables and indicator definitions. https://digital.nhs.uk/data-and-information/publications/statistical/quality-and-outcomes-framework-achievement-prevalence-and-exceptions-data/quality-and-outcomes-framework-2006-07-national-level

18 NHS Digital. Quality and Outcomes Framework (QOF) 2012-13. Prevalence data tables and indicator definitions. https://digital.nhs.uk/data-and-information/publications/statistical/quality-and-outcomes-framework-achievement-prevalence-and-exceptions-data/quality-and-outcomes-framework-2012-13

19 British Heart Foundation (BHF). Heart and Circulatory Disease Statistics 2022.

20 Go AS, Mozaffarian D, Roger VL, et al. Heart Disease and Stroke Statistics--2013 Update: A Report From the American Heart Association. Circulation 2013; 127: e6–245.

21 Tsao CW, Aday AW, Almarzooq ZI, et al. Heart Disease and Stroke Statistics—2023 Update: A Report From the American Heart Association. Circulation 2023; 147: e93–621.

22 Roth GA, Mensah GA, Johnson CO, et al. Global Burden of Cardiovascular Diseases and Risk Factors, 1990–2019: Update From the GBD 2019 Study. Journal of the American College of Cardiology 2020; 76: 2982–3021.

23 Institute for Health Metrics and Evaluation. Gobal Burden of Diseases Viz Hub. 2023. https://vizhub.healthdata.org/gbd-compare/ (accessed July 1, 2023).

24 Bossone E, Eagle KA. Epidemiology and management of aortic disease: aortic aneurysms and acute aortic syndromes. Nat Rev Cardiol 2021; 18: 331–48.

25 Sampson UKA, Norman PE, Fowkes FGR, et al. Estimation of Global and Regional Incidence and Prevalence of Abdominal Aortic Aneurysms 1990 to 2010. Global Heart 2014; 9: 159–70.

26 Chugh SS, Havmoeller R, Narayanan K, et al. Worldwide Epidemiology of Atrial Fibrillation. Circulation 2014; 129: 837–47.

27 Heit JA. Epidemiology of venous thromboembolism. Nat Rev Cardiol 2015; 12: 464–74.

28 Wendelboe AM, Raskob GE. Global Burden of Thrombosis. Circulation Research 2016; 118: 1340–7.

29 National Cardiac, Audit Programme. British Heart Rythm Society. National Audit of Cardiac Rhythm Management (NACRM). Summary report 2023. https://www.nicor.org.uk/national-cardiac-audit-programme/cardiac-rhythm-audit-nacrm

Supplementary text S3: Examples of disease-specific sensitivity analyses investigating the validity of disease definitions

In addition to the sensitivity analyses assessing the overall robustness and validity of disease definitions (**Supplementary text S1**), we also performed a series of disease-specific investigations into the validity of recorded diagnoses. Below we present two examples of such analyses.

To confirm the validity of heart failure cases included in our cohort, we performed the following sensitivity analyses. (a) case identification restricted to diagnostic codes included in national care monitoring programmes. While for our main analysis we intentionally expanded the diagnostic codes from the national audit programmes list with additional codes indicating a heart failure diagnosis, so as to ensure completeness; sensitivity analyses, restricting diagnostic codes to those used in the national audit programmes, found that 97% of patients in our cohort had a record heart failure used in the national clinical audit programmes, and led to no significant changes in the present results. (b) case identification restricted to diagnoses recorded in secondary care, or referred for specialist assessment or for echocardiography. We further found that 92% of patients included in our cohort had a heart failure diagnosis recorded in secondary care, or either a referral to specialist cardiology service or echocardiography. Sensitivity analyses using these more restrictive disease definitions led to modestly lower estimates of disease incidence, but no significant change in trends over time or difference by subgroups of age, sex, and socioeconomic status.

To confirm the validity of heart block diagnoses included in our cohort, we performed the following sensitivity analyses. (a) case identification restricted to diagnoses recorded during a hospital admission. These showed that 85% of patients with heart block also had a diagnosis of heart block recorded by specialists during a hospital admission. (b) case identification restricted to patients with a pacemaker implantation. These showed that 83% of individuals with heart block included in our study also had a pacemaker implanted. These findings are in line with expert expectations for this condition, as most but not all individuals with second or third degree heart block will require a pacemaker. Sensitivity analyses using these more restrictive disease definitions led to modestly lower estimates of disease incidence, but no significant change in trends over time or difference by subgroups of age, sex, and socioeconomic status.

Figure S1: Temporal trends in age at first diagnosis of cardiovascular diseases

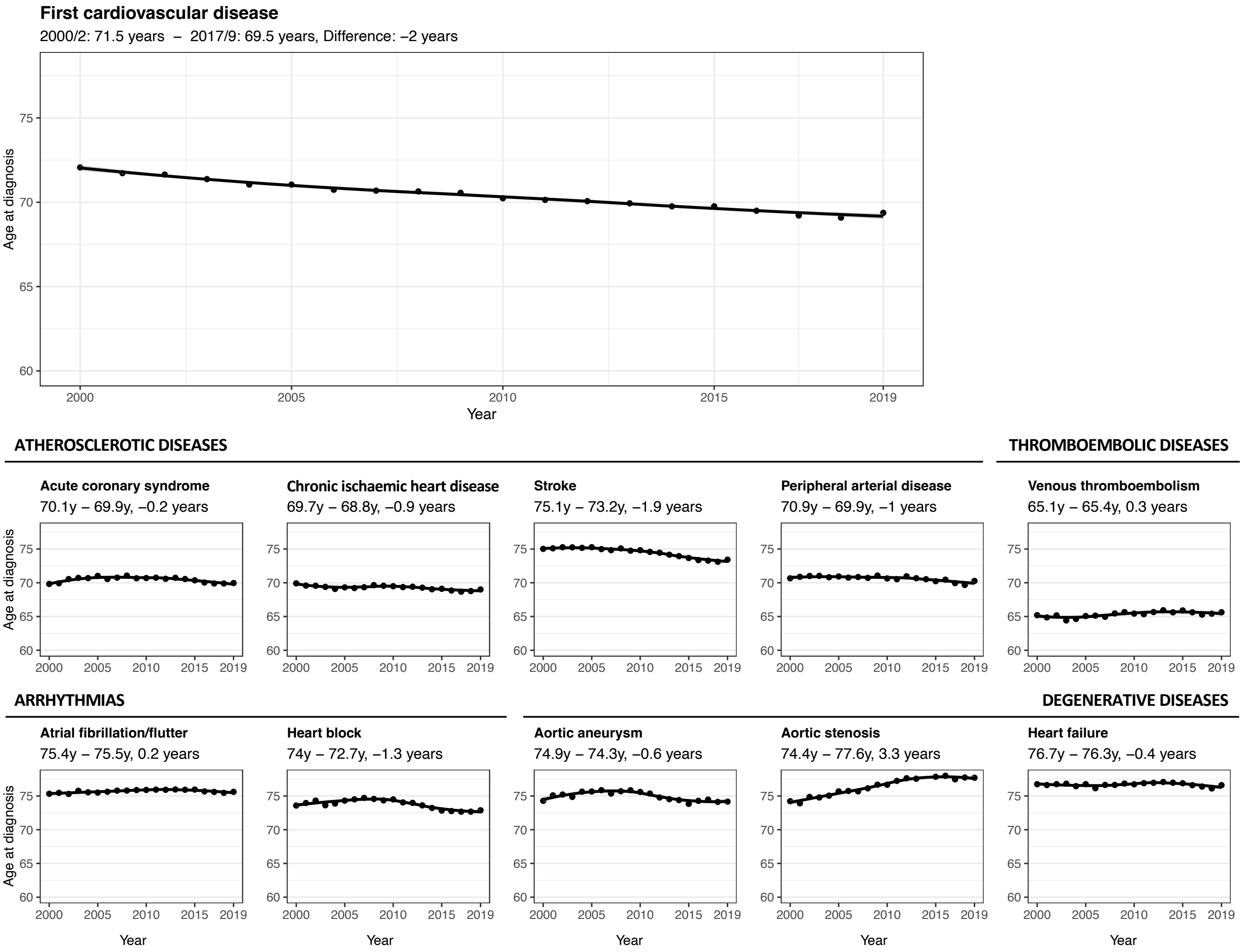

Mean age at diagnosis between 2000 and 2019. 'First cardiovascular disease' refers to the primary incidence of cardiovascular disease across the 10 conditions investigated in this study (that is the number of patients first diagnosed with a cardiovascular disease). Yearly estimates were smoothed using loess (locally estimated scatterplot smoothing) regression lines.

Figure S2: Incidence of cardiovascular diseases by region

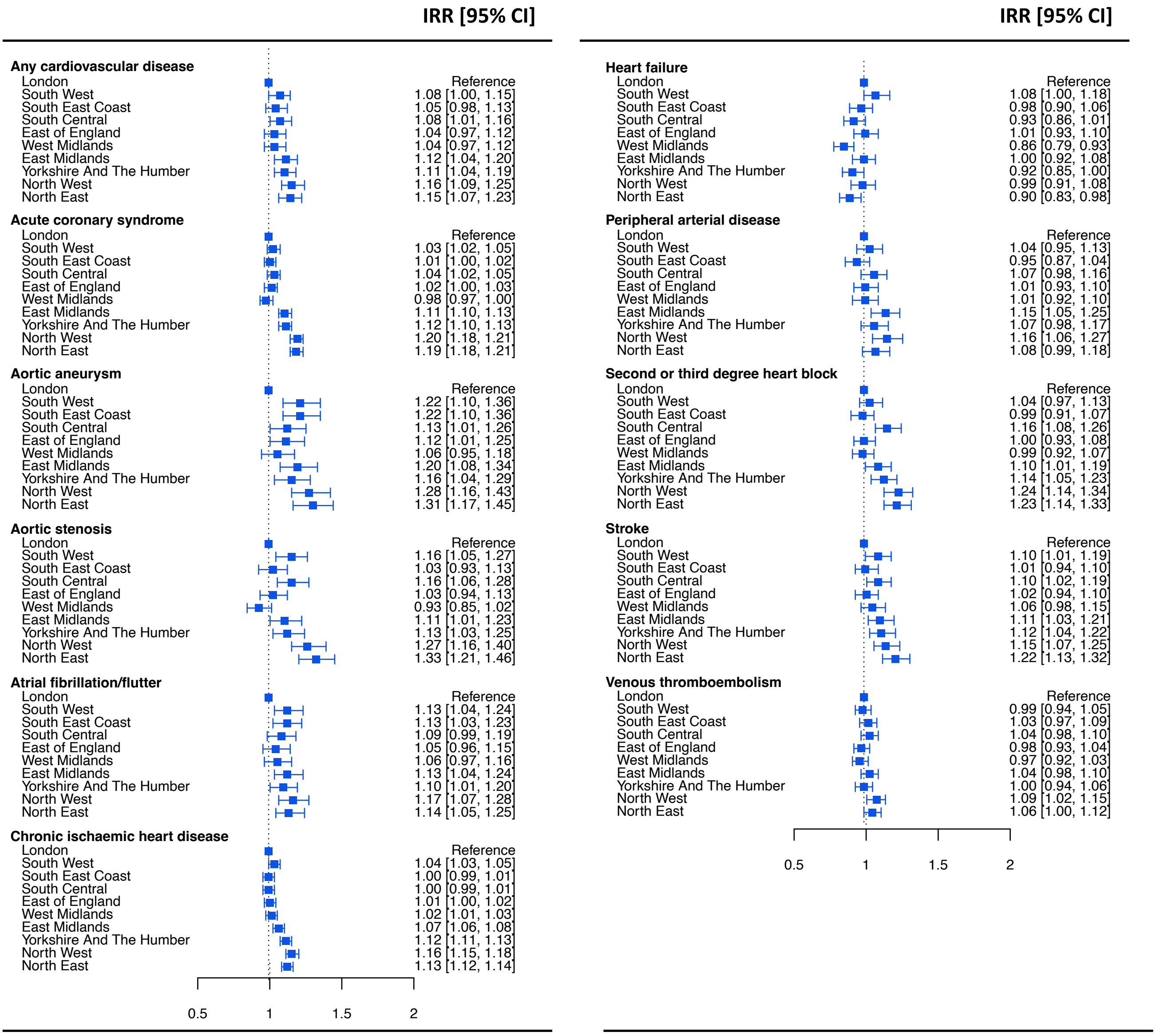

Age-sex-standardised incidence rate ratio by English region, adjusted for calendar year and socioeconomic status. 'Any cardiovascular disease' refers to the primary incidence of cardiovascular disease across the 10 conditions investigated in this study (that is the number of patients first diagnosed with a cardiovascular disease). London was set as the reference region and other regions are presented from lowest to highest latitude (South to North). IRR = Incidence Rate Ratio, 95% CI = 95% Confidence Interval.

Figure S3: Incidence of cardiovascular diseases over time from 2000-2019. Sensitivity analyses based on broader disease definitions.

Disease	N: All years 2000-2019	Standardised incidence per 100,000 person-years: 2000-2002	Standardised incidence per 100,000 person-years: 2017-2019	Temporal trend: IRR [95% CI] 2017-2019 vs. 2000-2002
Acute coronary syndrome	315 359	272	190	0.70 [0.69, 0.70]
	317 007	275	190	0.69 [0.68, 0.70]
Aortic aneurysm	78 060	46	64	1.38 [1.21, 1.58]
	78 076	46	64	1.38 [1.21, 1.58]
Aortic stenosis	99 081	41	98	2.42 [2.13, 2.74]
	108 176	45	107	2.36 [2.11, 2.64]
Atrial fibrillation/flutter	562 996	350	478	1.36 [1.20, 1.53]
	562 996	350	478	1.36 [1.20, 1.53]
Chronic ischaemic heart disease	539 807	514	351	0.67 [0.66, 0.67]
	539 807	514	351	0.67 [0.66, 0.67]
Heart failure	437 326	347	367	1.05 [0.94, 1.18]
	454 893	353	389	1.10 [0.98, 1.23]
Peripheral arterial disease	207 187	166	144	0.89 [0.80, 0.98]
	211 014	167	147	0.90 [0.81, 0.99]
Second or third degree heart block	141 375	64	149	2.22 [1.99, 2.46]
	160 867	70	176	2.44 [2.20, 2.70]
Stroke	292 694	242	181	0.75 [0.67, 0.83]
	301 836	246	190	0.77 [0.69, 0.85]
Venous thromboembolism or pulmonary embolism	232 885	141	183	1.28 [1.18, 1.39]
	232 885	141	183	1.28 [1.18, 1.39]
**Any cardiovascular disease**	**1 650 052**	**1 686**	**1 353**	**0.80 [0.73, 0.88]**
	**1 662 652**	**1 701**	**1 378**	**0.81 [0.74, 0.89]**

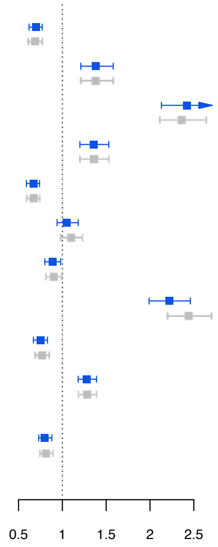

Main analyses
Sensitivity analyses, based on broader disease definitions

Incidence rates are presented as incidence rates per 100 000 person-years at risk and are age-sex-standardised to the 2013 European Standard Population. 'Any cardiovascular disease' refers to the primary incidence of cardiovascular disease across the 10 conditions investigated in this study (that is the number of patients first diagnosed with a cardiovascular disease). 'N' refers to the number of patients newly diagnosed with cardiovascular disease during the study period. Main analyses are presented in blue. Sensitivity analyses (in grey) included a broader set of diagnostic codes in disease definitions. For list of diagnostic codes used in main and sensitivity analyses see Table S2. IRR = Incidence Rate Ratio, 95% CI = 95% Confidence Interval.

Figure S4: Incidence of cardiovascular diseases over time from 2000-2019. Sensitivity analyses including diagnoses recorded on death certificates

	N	Standardised incidence per 100,000 person-years		Temporal trend
**Disease**	**All years** 2000-2019	**2000-2002**	**2017-2019**	**IRR [95% CI]** 2017-2019 vs. 2000-2002
Acute coronary syndrome	315 359<br>358 261	272<br>323	190<br>208	0.70 [0.69, 0.70]<br>0.64 [0.63, 0.65]
Aortic aneurysm	78 060<br>84 999	46<br>55	64<br>66	1.38 [1.21, 1.58]<br>1.20 [1.05, 1.38]
Aortic stenosis	99 081<br>102 066	41<br>43	98<br>100	2.42 [2.13, 2.74]<br>2.33 [2.06, 2.65]
Atrial fibrillation/flutter	562 996<br>565 905	350<br>354	478<br>480	1.36 [1.20, 1.53]<br>1.35 [1.19, 1.52]
Chronic ischaemic heart disease	539 807<br>628 208	514<br>608	351<br>393	0.67 [0.66, 0.67]<br>0.64 [0.63, 0.64]
Heart failure	437 326<br>472 859	347<br>387	367<br>382	1.05 [0.94, 1.18]<br>0.98 [0.88, 1.11]
Peripheral arterial disease	207 187<br>210 724	166<br>170	144<br>146	0.89 [0.80, 0.98]<br>0.87 [0.79, 0.97]
Second or third degree heart block	141 375<br>141 561	64<br>64	149<br>149	2.22 [1.99, 2.46]<br>2.21 [1.98, 2.46]
Stroke	292 694<br>318 883	242<br>274	181<br>191	0.75 [0.67, 0.83]<br>0.70 [0.63, 0.78]
Venous thromboembolism or pulmonary embolism	232 885<br>249 511	141<br>155	183<br>191	1.28 [1.18, 1.39]<br>1.22 [1.12, 1.32]
**Any cardiovascular disease**	**1 650 052**<br>**1 763 969**	**1 686**<br>**1 848**	**1 353**<br>**1 412**	**0.80 [0.73, 0.88]**<br>**0.76 [0.69, 0.84]**

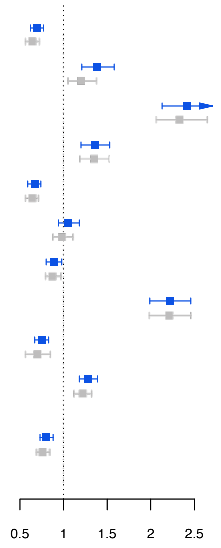

Excluding diagnoses recorded on death certificates
Including diagnoses recorded on death certificates

Incidence rates are presented as incidence rates per 100 000 person-years at risk and are age-sex-standardised to the 2013 European Standard Population. 'Any cardiovascular disease' refers to the primary incidence of cardiovascular disease across the 10 conditions investigated in this study (that is the number of patients first diagnosed with a cardiovascular disease). 'N' refers to the number of patients newly diagnosed with cardiovascular disease during the study period. Main analyses (in blue) refer to diagnoses recorded in primary or secondary care. Sensitivity analyses (in grey) refer to diagnoses recorded in primary care, secondary care or death certificates. IRR = Incidence Rate Ratio, 95% CI = 95% Confidence Interval.

Figure S5: Incidence of cardiovascular diseases over time from 2000-2019. Sensitivity analyses with longer lookback period

Disease	N<br>All years 2000-2019	Standardised incidence per 100,000 person-years<br>2000-2002	 2017-2019	Temporal trend<br>IRR [95% CI] 2017-2019 vs. 2000-2002
Acute coronary syndrome	315 359<br>249 070	272<br>270	190<br>190	0.70 [0.69, 0.70]<br>0.71 [0.70, 0.72]
Aortic aneurysm	78 060<br>65 813	46<br>52	64<br>64	1.38 [1.21, 1.58]<br>1.23 [1.07, 1.41]
Aortic stenosis	99 081<br>85 325	41<br>51	98<br>100	2.42 [2.13, 2.74]<br>1.97 [1.72, 2.24]
Atrial fibrillation/flutter	562 996<br>467 438	350<br>372	478<br>482	1.36 [1.20, 1.53]<br>1.28 [1.13, 1.46]
Chronic ischaemic heart disease	539 807<br>416 583	514<br>434	351<br>353	0.67 [0.66, 0.67]<br>0.81 [0.80, 0.81]
Heart failure	437 326<br>350 433	347<br>317	367<br>369	1.05 [0.94, 1.18]<br>1.18 [1.04, 1.33]
Peripheral arterial disease	207 187<br>163 930	166<br>164	144<br>144	0.89 [0.80, 0.98]<br>0.89 [0.80, 0.99]
Second or third degree heart block	141 375<br>119 906	64<br>71	149<br>150	2.22 [1.99, 2.46]<br>2.05 [1.84, 2.30]
Stroke	292 694<br>230 486	242<br>235	181<br>180	0.75 [0.67, 0.83]<br>0.77 [0.69, 0.86]
Venous thromboembolism or pulmonary embolism	232 885<br>185 473	141<br>142	183<br>183	1.28 [1.18, 1.39]<br>1.26 [1.16, 1.37]
**Any cardiovascular disease**	**1 650 052**<br>**1 261 354**	**1 686**<br>**1 510**	**1 353**<br>**1 340**	**0.80 [0.73, 0.88]**<br>**0.89 [0.80, 0.98]**

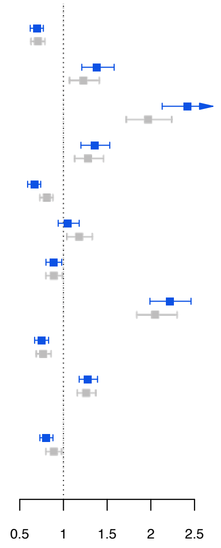

Lookback period 1 year
Lookback period 4 years

Incidence rates are presented as incidence rates per 100 000 person-years at risk and are age-sex-standardised to the 2013 European Standard Population. 'Any cardiovascular disease' refers to the primary incidence of cardiovascular disease across the 10 conditions investigated in this study (that is the number of patients first diagnosed with a cardiovascular disease). 'N' refers to the number of patients newly diagnosed with cardiovascular disease during the study period. Main analyses (in blue) use a lookback period of 1 year to exclude possibly prevalent cases (ie. individuals with a first diagnosis of that condition during the first 12 months of registering with their general practitioner are excluded from incidence calculations). Sensitivity analyses (in grey) use a lookback period of 4 years. IRR = Incidence Rate Ratio, 95% CI = 95% Confidence Interval.

Figure S6: Incidence of cardiovascular diseases over time from 2000-2019. Sensitivity analyses restricted to diagnoses recorded during hospital admissions.

Disease	N<br>All years<br>2000-2019	Standardised incidence per 100,000 person-years<br>2000-2002	 2017-2019	Temporal trend<br>IRR [95% CI]<br>2017-2019 vs. 2000-2002
Acute coronary syndrome	315 359	272	190	0.70 [0.69, 0.70]
	278 995	214	173	0.79 [0.79, 0.80]
Aortic aneurysm	78 060	46	64	1.38 [1.21, 1.58]
	61 095	30	55	1.89 [1.65, 2.17]
Aortic stenosis	99 081	41	98	2.42 [2.13, 2.74]
	75 628	24	79	3.19 [2.79, 3.65]
Atrial fibrillation/flutter	562 996	350	478	1.36 [1.20, 1.53]
	495 463	241	446	1.83 [1.61, 2.07]
Chronic ischaemic heart disease	539 807	514	351	0.67 [0.66, 0.67]
	473 409	275	313	1.11 [1.10, 1.12]
Heart failure	437 326	347	367	1.05 [0.94, 1.18]
	368 748	217	334	1.52 [1.35, 1.71]
Peripheral arterial disease	207 187	166	144	0.89 [0.80, 0.98]
	144 034	80	124	1.58 [1.43, 1.75]
Second or third degree heart block	141 375	64	149	2.22 [1.99, 2.46]
	119 975	55	123	2.13 [1.90, 2.38]
Stroke	292 694	242	181	0.75 [0.67, 0.83]
	218 577	151	140	0.92 [0.82, 1.02]
Venous thromboembolism or pulmonary embolism	232 885	141	183	1.28 [1.18, 1.39]
	154 549	90	127	1.40 [1.29, 1.52]
**Any cardiovascular disease**	**1 650 052**	**1 686**	**1 353**	**0.80 [0.73, 0.88]**
	**1 382 000**	**1 284**	**1 132**	**0.89 [0.80, 0.98]**

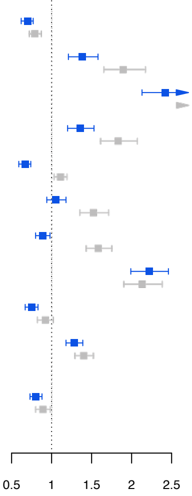

Main analyses
Sensitivity analyses, restricted to diagnoses recorded during a hospital admission

Incidence rates are presented as incidence rates per 100 000 person-years at risk and are age-sex-standardised to the 2013 European Standard Population. 'Any cardiovascular disease' refers to the primary incidence of cardiovascular disease across the 10 conditions investigated in this study (that is the number of patients first diagnosed with a cardiovascular disease). 'N' refers to the number of patients newly diagnosed with cardiovascular disease during the study period. IRR = Incidence Rate Ratio, 95% CI = 95% Confidence Interval.

Figure S7: Initiation of cardiovascular prevention therapy within 6 months of diagnosis, among patients diagnosed with cardiovascular disease in the periods 2000-2002 and 2017-2019.

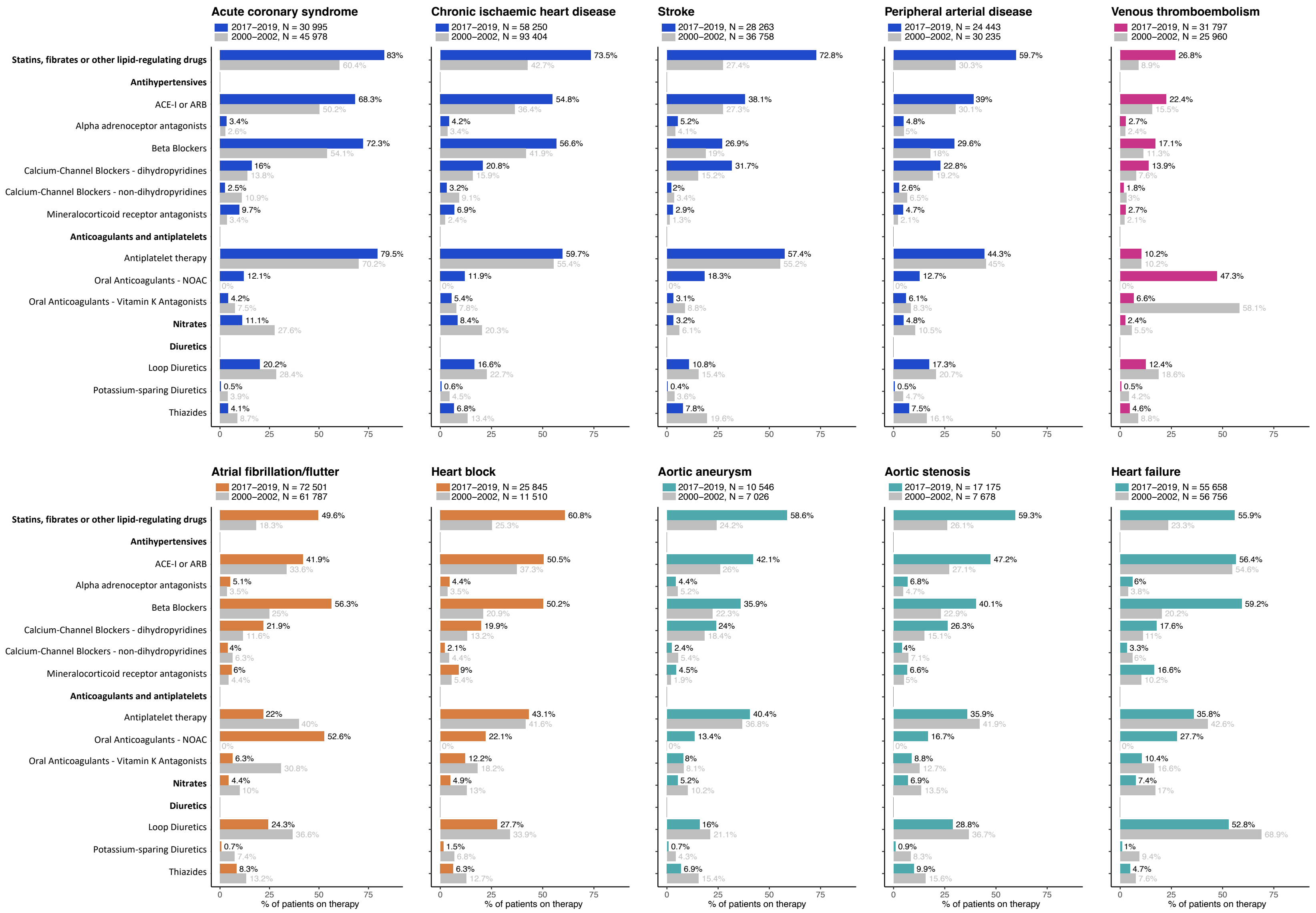

Cardiovascular prevention therapies refer to the percentage of patients with at least 2 prescriptions within 6 months after incident cardiovascular disease (CVD). Analyses were restricted to patients alive and registered with a general practice 30 days after diagnosis. ACE- inhibitors = Angiotensin-Converting Enzyme Inhibitors, ARB = Angiotensin II Receptor Blockers, NOAC = Non-vitamin K Antagonist Oral Anticoagulant. Detailed list of drug substances included in each drug class are presented in ***Table S2****.*

Table S1: Clinical codes used to define cardiovascular diseases

For each condition, a list of diagnostic codes from was compiled to identify diagnoses based on the coding schemes in use in each data source (International Classification of Diseases, tenth revision (ICD-10) for diagnoses recorded in secondary care; ICD-9 (in use until 31/12/2000) and ICD-10 for diagnoses recorded on death certificates (used in sensitivity analyses only); UK Office of Population Census and Surveys classification (OPCS-4) for procedures performed in secondary care settings; and CPRD Aurum and CPRD Gold code dictionaries for primary care data, which include a combination of Read, SNOMED, and local EMIS codes.[25]

The clinical code lists used to identify individual conditions presented in this manuscript are accessible in a machine-readable format (as a tab-delimited text file) on our GitHub repository (see https://github.com/nathalieconrad/CVD_incidence).

Codes used in sensitivity analyses referring to broader disease definitions are labelled accordingly.

Note: Diagnostic codes should be imported, stored, and processed as text rather than integers to avoid automatic reformatting of long-digit numbers by certain software packages.

Table S2: Drug class definitions

Drug class	Definition
Angiotensin-Converting Enzyme Inhibitors (ACE-I)	BNF chapter 02050501 “Angiotensin-Converting Enzyme Inhibitors”
Alpha adrenoceptor antagonists	BNF chapter 02050400 “Alpha-Adrenoceptor Blockers” and Terazosin hydrochloride
Antiplatelet therapy	BNF chapter 02090000 “Antiplatelet Dugs”
Angiotensin II Receptor Blockers (ARB)	BNF chapter 02050502 “Angiotensin-II Receptor Antagonists”
Beta blockers	BNF chapters 02040000 “Beta-Adrenoceptor Blockers” and 02040100 “Beta-Blockers With Diuretics” (excluding eye drops)
Calcium-channel blockers (dihydropyridines)	Amlodipine, Felodipine, Isradipine, Lacidipine, Lercanidipine hydrochloride, Nicardipine hydrochloride, Nifedipine, Nimodipine, and Nisoldipine
Calcium-channel blockers (non-dihydropyridines)	Diltiazem hydrochloride and Verapamil hydrochloride (excluding creams and ointments)
Statins and other lipid-regulating drugs	BNF chapter 02120400 “Statins”, 02120300 “Fibrates”, 02120200 ”Ezetimibe”, 02120000 “Lipid-Regulating Drugs”, 02120700 “Other Lipid-regulating Drugs”
Loop diuretics	BNF chapter 2020200 “Loop Diuretics” and mixed preparations containing Bumetanide or Furosemide
Mineralocorticoid receptor antagonists	Eplerenone and Spironolactone
Nitrates	BNF chapter 02060100 “Nitrates” (excluding creams, ointments, sublingual tablets, buccal tablets and sprays)
Oral Anticoagulants - NOAC	Apixaban, Dabigatran etexilate mesilate, Edoxaban tosilate, Rivaroxaban
Oral Anticoagulants - Vitamin K Antagonists	Acenocoumarol, Phenindione, Phenprocoumon, Warfarin sodium
Parenteral anticoagulants	BNF chapter 02080100 Parenteral Anticoagulants
Potassium-sparing diuretics	BNF chapter 02020400 “Potassium-Sparing Diuretics With Other Diuretics” and mixed preparations containing Amiloride or Triamterene
Sacubitril/Valsartan	Sacubitril/Valsartan
Thiazides	BNF chapter 02020100 “Thiazides And Related Diuretics”.

BNF = British National Formulary. A list of product/brand names was compiled based on the above definitions, so as to ensure a comprehensive extraction of drug prescriptions that includes drug dictionary entries with missing drug substance or BNF chapter reference.

Table S3: Characteristics of patients with incident cardiovascular disease between 2000 and 2019, stratified by age at diagnosis

	All patients (N = 1 650 052)	Age at diagnosis	
		Less than 60 years (N = 352 626)	More than 60 years (N = 129 7426)
**Women, N (%)**	784 904 (47.6%)	133 549 (37.9%)	651 355 (50.2%)
**Ethnicity**			
African/Caribbean	25 518 (1.6%)	12 595 (1.7%)	12 923 (1.6%)
Asian	41 704 (2.6%)	17 418 (2.3%)	24 286 (2.9%)
Mixed/Other	33 582 (2.1%)	14 163 (1.9%)	19 419 (2.3%)
White	1 480 577 (93.6%)	705 549 (94.1%)	775 028 (93.2%)
Missing	68 671 (4.2%)	35 179 (4.5%)	33 492 (3.9%)
**Socioeconomic status quintile**			
1 (least deprived)	353 985 (21.5%)	64 532 (18.3%)	289 453 (22.3%)
2	344 196 (20.9%)	64 365 (18.3%)	279 831 (21.6%)
3	336 564 (20.4%)	67 039 (19.0%)	269 525 (20.8%)
4	316 077 (19.2%)	73 262 (20.8%)	242 815 (18.7%)
5 (most deprived)	299 230 (18.1%)	83 428 (23.7%)	215 802 (16.6%)
**Systolic blood pressure** (mmHg)			
Mean (SD)	138 (20.2)	133 (19.6)	139 (20.2)
Missing (%)	256 402 (15.5%)	88 827 (25.2%)	167 575 (12.9%)
**Diastolic blood pressure** (mmHg)			
Mean (SD)	80.0 [70.0, 85.0]	80.0 [74.0, 88.0]	79.0 [70.0, 84.0]
Missing (%)	256 952 (15.6%)	88 894 (25.2%)	168 058 (13.0%)
**Body mass index category**			
Underweight	22 826 (2.9%)	3 119 (2.0%)	19 707 (3.1%)
Normal weight	228 699 (28.6%)	34 861 (21.9%)	193 838 (30.2%)
Overweight	287 902 (36.0%)	51 116 (32.0%)	236 786 (36.9%)
Obesity	261 058 (32.6%)	70 423 (44.1%)	190 635 (29.7%)
Missing (%)	849 567 (51.5%)	193 107 (54.8%)	656 460 (50.6%)
**Smoking status**			
Yes	226 019 (21.3%)	83 101 (37.2%)	142 918 (17.0%)
Ex	353 276 (33.3%)	51 740 (23.2%)	301 536 (35.9%)
No	482 768 (45.5%)	88 276 (39.6%)	394 492 (47.0%)
Missing (%)	587 989 (35.6%)	129 509 (36.7%)	458 480 (35.3%)
**Cholesterol** (total cholesterol/high-density lipoprotein ratio)			
Mean (SD)	3.73 (1.26)	4.32 (1.43)	3.60 (1.17)
Missing (%)	1 049 256 (63.6%)	244 382 (69.3%)	804 874 (62.0%)
**Comorbidities**			
Chronic kidney disease	296 554 (18.0%)	51 961 (14.7%)	244 593 (18.9%)
Dyslipidaemia	820 892 (49.7%)	106 054 (30.1%)	714 838 (55.1%)
Hypertension	233 833 (14.2%)	37 929 (10.8%)	195 904 (15.1%)
Type 2 diabetes	233 833 (14.2%)	103 260 (13.2%)	130 573 (15.1%)

Patient characteristics at the time of their first cardiovascular disease diagnosis. Socioeconomic status was defined as the Index of Multiple Deprivation (IMD) 2015 quintile, with SES 1 referring to the most affluent and SES 5 to the most deprived socioeconomic quintile. Blood pressure, body mass index, smoking status and cholesterol are presented as the latest measurement within two years prior to first cardiovascular disease (CVD) diagnosis. Comorbidities are presented as the percentage of patients diagnosed with the condition of interest at any time up first CVD diagnosis. Number and percentage of records with missing data are displayed for variables with missing entries. For variables with missing entries, summary statistics present observed values alongside the percentage of missing values. Category percentages refer to complete cases.

Table S4: Crude incidence rates of individual cardiovascular diseases, stratified by age and sex, for the period 2017-2019

Acute coronary syndrome

Age band	Men and women	Women	Men
0-24 years	0.9	0.6	1.3
25-29 years	4.9	2.8	7.0
30-34 years	10.3	5.6	14.8
35-39 years	20.6	11.7	29.3
40-44 years	53.6	26.5	79.5
45-49 years	105.1	58.2	150.3
50-54 years	179.1	98.2	257.8
55-59 years	249.5	135.5	361.3
60-64 years	336.3	192.8	481.5
65-69 years	396.7	252.7	550.6
70-74 years	493.5	348.0	655.5
75-79 years	648.5	506.0	819.4
80-84 years	834.8	723.2	981.2
85-89 years	1123.4	996.5	1320.9
90+ years	1461.6	1359.9	1686.3

Aortic aneurysm

Age band	Men and women	Women	Men
0-24 years	1.2	0.9	1.5
25-29 years	2.3	1.5	3.0
30-34 years	3.0	2.3	3.6
35-39 years	3.7	2.4	5.0
40-44 years	7.0	4.3	9.7
45-49 years	9.2	4.9	13.3
50-54 years	15.9	9.2	22.3
55-59 years	28.3	12.3	43.7
60-64 years	63.5	19.7	106.8
65-69 years	188.6	44.1	340.4
70-74 years	174.4	88.1	268.5
75-79 years	290.8	140.1	468.1
80-84 years	378.5	204.8	604.0
85-89 years	460.0	277.8	741.6
90+ years	468.7	309.6	819.9

Aortic stenosis

Age band	Men and women	Women	Men
0-24 years	2.3	1.6	3.0
25-29 years	1.9	2.2	1.6
30-34 years	2.3	2.4	2.1
35-39 years	3.3	2.8	3.7
40-44 years	6.4	5.7	7.0
45-49 years	10.4	8.8	12.0
50-54 years	18.2	15.3	21.0
55-59 years	40.3	26.6	53.4
60-64 years	76.9	56.1	97.4
65-69 years	134.5	102.4	167.7
70-74 years	251.4	194.5	312.8
75-79 years	455.2	385.2	536.4

80-84 years	737.2	620.5	886.3
85-89 years	1069.7	961.6	1233.5
90+ years	1266.0	1133.9	1552.0

Atrial fibrillation/flutter

Age band	Men and women	Women	Men
0-24 years	3.5	2.6	4.3
25-29 years	13.8	9.3	18.0
30-34 years	19.4	14.0	24.7
35-39 years	26.7	19.6	33.5
40-44 years	46.7	33.9	58.9
45-49 years	88.7	57.2	118.9
50-54 years	152.0	98.4	203.9
55-59 years	267.5	183.6	349.1
60-64 years	470.0	320.6	619.8
65-69 years	815.3	593.3	1051.6
70-74 years	1281.1	970.8	1628.1
75-79 years	2077.4	1668.4	2574.1
80-84 years	3150.5	2748.8	3689.9
85-89 years	4454.8	3996.8	5192.3
90+ years	6056.1	5595.4	7118.3

Chronic ischaemic heart disease

Age band	Men and women	Women	Men
0-24 years	1.6	1.0	2.2
25-29 years	7.5	5.7	9.3
30-34 years	15.1	12.4	17.7
35-39 years	33.0	18.9	46.6
40-44 years	88.2	52.2	122.5
45-49 years	178.3	115.0	239.2
50-54 years	312.3	196.5	425.3
55-59 years	480.1	303.8	654.3
60-64 years	670.7	431.7	916.3
65-69 years	828.9	569.3	1112.8
70-74 years	1022.0	744.2	1340.4
75-79 years	1312.6	1045.8	1643.3
80-84 years	1531.6	1291.5	1857.2
85-89 years	1649.8	1452.4	1967.9
90+ years	1676.8	1535.6	1997.4

Heart block

Age band	Men and women	Women	Men
0-24 years	7.1	7.4	6.9
25-29 years	9.1	8.2	10.0
30-34 years	12.2	10.8	13.5
35-39 years	15.5	15.1	16.0
40-44 years	22.1	16.2	27.7
45-49 years	35.2	24.7	45.2
50-54 years	65.5	39.7	90.4
55-59 years	104.9	56.1	152.1

60-64 years	171.8	88.8	254.3
65-69 years	265.5	156.8	379.3
70-74 years	425.5	250.9	616.1
75-79 years	664.7	431.5	940.5
80-84 years	938.8	644.1	1325.4
85-89 years	1088.5	746.2	1628.4
90+ years	1082.4	789.3	1747.7

Heart failure

Age band	Men and women	Women	Men
0-24 years	6.1	4.9	7.3
25-29 years	15.2	13.6	16.7
30-34 years	21.0	19.0	23.0
35-39 years	26.9	24.0	29.6
40-44 years	44.3	34.6	53.5
45-49 years	76.3	55.1	96.7
50-54 years	131.6	88.4	173.3
55-59 years	206.9	147.6	264.4
60-64 years	329.2	237.5	420.6
65-69 years	529.6	390.5	676.1
70-74 years	819.2	644.7	1010.5
75-79 years	1462.7	1236.7	1730.5
80-84 years	2393.2	2116.0	2754.0
85-89 years	3871.7	3477.3	4484.3
90+ years	5722.2	5292.0	6670.6

Peripheral arterial disease

Age band	Men and women	Women	Men
0-24 years	7.2	6.2	8.2
25-29 years	12.8	12.8	12.8
30-34 years	18.3	16.8	19.7
35-39 years	25.4	22.5	28.2
40-44 years	33.9	29.2	38.4
45-49 years	65.4	54.0	76.3
50-54 years	93.7	72.0	114.6
55-59 years	144.7	100.1	187.8
60-64 years	228.6	149.1	307.8
65-69 years	303.7	208.0	404.3
70-74 years	396.9	298.6	504.4
75-79 years	550.5	416.2	709.3
80-84 years	710.4	556.2	910.1
85-89 years	884.6	758.7	1078.4
90+ years	1009.4	929.6	1184.1

Stroke

Age band	Men and women	Women	Men
0-24 years	5.2	4.2	6.1
25-29 years	9.1	7.6	10.6
30-34 years	14.6	15.5	13.7

35-39 years	22.6	21.3	23.8
40-44 years	40.0	33.9	45.8
45-49 years	62.5	52.2	72.4
50-54 years	102.9	78.5	126.4
55-59 years	152.5	110.0	193.5
60-64 years	226.0	165.8	285.8
65-69 years	314.1	249.6	381.5
70-74 years	440.1	369.5	516.8
75-79 years	709.5	642.2	788.2
80-84 years	1070.6	995.9	1166.5
85-89 years	1549.2	1516.6	1598.8
90+ years	2091.2	2146.0	1972.5

Venous thromboembolism

Age band	Men and women	Women	Men
0-24 years	11.8	16.1	7.8
25-29 years	53.2	73.6	34.1
30-34 years	66.4	84.0	49.5
35-39 years	80.8	94.7	67.4
40-44 years	102.0	108.4	96.0
45-49 years	130.4	130.0	130.8
50-54 years	162.1	154.9	169.0
55-59 years	210.5	182.8	237.3
60-64 years	265.7	236.0	295.2
65-69 years	361.1	325.9	397.7
70-74 years	464.5	440.2	490.7
75-79 years	611.5	599.8	625.0
80-84 years	740.6	757.4	719.5
85-89 years	892.7	930.8	835.9
90+ years	1032.3	1074.2	943.1

Crude incidence rates per 100 000 person-years at risk, stratified by five-year age-band and sex. Age groups between 0 and 24 years were grouped together due to low incidence of cardiovascular disease in these age groups. Incidence rates refer to the period 2017-2019.
